# Operant conditioning of spinal reflexes: from basic science to clinical therapy

**DOI:** 10.3389/fnint.2014.00025

**Published:** 2014-03-18

**Authors:** Aiko K. Thompson, Jonathan R. Wolpaw

**Affiliations:** ^1^Helen Hayes Hospital, New York State Department of HealthWest Haverstraw, NY, USA; ^2^Wadsworth Center, New York State Department of HealthAlbany, NY, USA; ^3^Department of Neurology, Neurological Institute, Columbia UniversityNew York, NY, USA; ^4^Department of Biomedical Sciences, University at Albany, State University of New YorkAlbany, NY, USA

**Keywords:** H-reflex, spinal cord plasticity, locomotion, learning and memory, spinal cord injury

## Abstract

New appreciation of the adaptive capabilities of the nervous system, recent recognition that most spinal cord injuries are incomplete, and progress in enabling regeneration are generating growing interest in novel rehabilitation therapies. Here we review the 35-year evolution of one promising new approach, operant conditioning of spinal reflexes. This work began in the late 1970’s as basic science; its purpose was to develop and exploit a uniquely accessible model for studying the acquisition and maintenance of a simple behavior in the mammalian central nervous system (CNS). The model was developed first in monkeys and then in rats, mice, and humans. Studies with it showed that the ostensibly simple behavior (i.e., a larger or smaller reflex) rests on a complex hierarchy of brain and spinal cord plasticity; and current investigations are delineating this plasticity and its interactions with the plasticity that supports other behaviors. In the last decade, the possible therapeutic uses of reflex conditioning have come under study, first in rats and then in humans. The initial results are very exciting, and they are spurring further studies. At the same time, the original basic science purpose and the new clinical purpose are enabling and illuminating each other in unexpected ways. The long course and current state of this work illustrate the practical importance of basic research and the valuable synergy that can develop between basic science questions and clinical needs.

## Introduction

Perhaps the most important neuroscience advance of the past half-century has been the recognition that the central nervous system (CNS) changes throughout life, that activity-dependent plasticity occurs continually everywhere in the CNS. The numerous ramifications of this recognition are now being pursued. Spinal cord research is a particularly active area. New appreciation of spinal cord plasticity, evidence that most spinal cord injuries are incomplete, and some success in initiating regeneration are encouraging the translation of basic science insights into new methods for improving recovery of function. For example, the benefits of locomotor training, which were first discovered in the 1950’s (Shurrager and Dykman, 1951) and drew renewed attention in the 1980’s (Lovely et al., 1986; Barbeau and Rossignol, 1987), have developed into an important rehabilitation method (Wernig and Müller, 1992; Edgerton et al., 2001, 2008; Maegele et al., 2002; Barbeau, 2003; Harkema et al., 2012). This review tracks the evolution of another basic science exploration of spinal cord plasticity into a promising new clinical therapy. It emphasizes the importance of basic science for clinical progress and the powerful synergy that can develop between basic and clinical research.

The concept of the spinal cord as a hard-wired reflex center, enunciated by Marshall Hall in the 19th century (Hall, 1833) and widely accepted through the 20th century, continues to exert strong influence, despite the extensive evidence for activity-dependent spinal cord plasticity in health and disease (Mendell, 1984; Wolpaw and Carp, 1993; Wolpaw and Tennissen, 2001; Edgerton et al., 2004; Frigon and Rossignol, 2006; Zehr, 2006; Petruska et al., 2007; Courtine et al., 2009; Dietz et al., 2009; Wolpaw, 2010; Rossignol et al., 2011; Grau, 2013). This evidence falls into two broad categories: plasticity in the spinal cord produced by physiological or pathological influence from the brain; and plasticity in the isolated spinal cord produced by peripheral input. Operant conditioning of spinal cord reflexes falls into the first category.

The first effort to demonstrate such conditioning was based on Anna Di Giorgio’s demonstration that abnormal descending activity caused by a supraspinal lesion could produce change in the spinal cord that persisted after the activity ended (Di Giorgio, 1929, 1942), and on evidence of task-dependent changes in spinal reflexes (reviewed in Wolpaw et al., 1983b). Taken together, these studies suggested that, if an operant conditioning protocol induced the brain to produce a constant descending influence on a spinal reflex pathway, and if that influence continued long enough, the spinal reflex pathway would change. The protocol would thereby create a unique model for studying the substrates of a memory (i.e., a persistent change in behavior) in the mammalian CNS (Wolpaw, 1982; Wolpaw et al., 1983b). The experimental accessibility and relative simplicity of the spinal cord, its connection to the brain by well-defined and accessible pathways, and its direct connection to behavior would make it possible to describe the spinal cord plasticity underlying the memory, understand exactly how it changes behavior, and define the brain-spinal cord interactions that create and maintain it.

**Figure 1:**
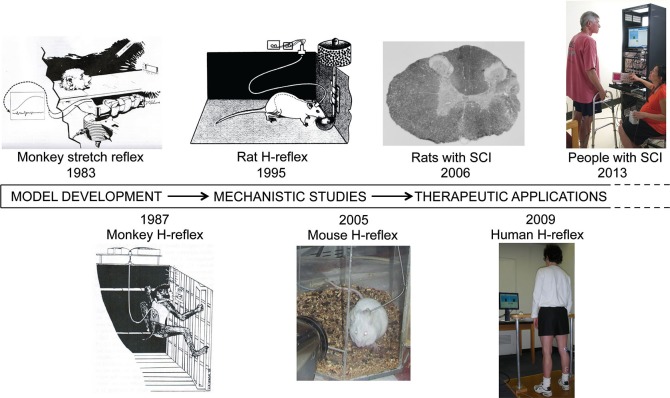
**Operant conditioning of spinal reflexes from 1978 to 2013.** The work began with model development and progressed to mechanistic studies and then to clinical applications. These three phases have overlapped to a considerable degree and continue to do so. (SCI: spinal cord injury) (Wolpaw et al., 1983b; Wolpaw, 1987; Chen and Wolpaw, 1995; Carp et al., 2006a; Chen et al., 2006d; Thompson et al., 2009, 2013b).

Since its beginning in 1978, this work has progressed through model development to mechanistic studies to clinical application. Figure 1 shows the major landmarks. As early mechanistic studies began to elucidate the mechanisms of conditioning, they encouraged pre-clinical and clinical studies, which in turn are now guiding further mechanistic investigations.

## Model development: establishing the phenomenon

In the late 1950’s, motor physiologists became very interested in the sequence of electromyographic (EMG) responses to sudden muscle stretch in monkeys and humans performing simisometric tasks, such as maintaining elbow angle against a constant opposing torque (e.g., Hammond, 1956; Lee and Tatton, 1975; Bawa et al., 1979; Lee et al., 1983). They identified a succession of responses that began with the earliest purely spinal and largely monosynaptic M1 response (i.e., the spinal stretch reflex (SSR)), proceeded through multisynaptic spinal or transcortical M2 and M3 components, and ended with late clearly intentional responses (Hammond, 1956; Lee and Tatton, 1975; Bawa et al., 1979; Lee et al., 1983; Christensen et al., 2000, 2001; Grey et al., 2001). The dependence of these components on prior instruction (i.e., “oppose the sudden stretch” or “do not oppose”) grew with their latency: the latest purely intentional responses were absent for the “do not oppose” instruction; middle-latency components were much reduced; and the earliest M1 component showed little or no effect.

In these studies, the instruction typically changed from trial to trial; and the investigators focused on the later components and their relationships to and dependence on neuronal activity in sensorimotor cortex (e.g., Evarts and Tanji, 1974). Nevertheless, some studies noted that the instruction had some effect on the earliest purely spinal M1 component (reviewed in Wolpaw et al., 1983b). This observation, combined with the Di Giorgio studies (Di Giorgio, 1929, 1942), prompted the initial effort to determine whether an operant conditioning protocol could change the SSR in the monkey biceps muscle (Wolpaw et al., 1983a). The protocol had three key features: (1) it required maintenance of both a certain elbow angle and a certain level of biceps EMG activity as the animal opposed a constant extension torque; (2) it based reward on the size of the SSR (measured by EMG) evoked by a brief pulse of additional extension torque; and (3) the reward criterion (i.e., equivalent to the instruction) remained constant over days and weeks (i.e., reward occurred when the SSR was above (up-conditioning) or below (down-conditioning) a criterion). In sum, the protocol was designed to induce and maintain a long-term change in descending influence over the spinal arc of the reflex, and to thereby change the spinal cord.

This protocol was successful: in 10 of 11 monkeys, biceps SSR size changed substantially in the correct direction, and lesser SSR changes occurred in the biceps synergists, brachialis and brachioradialis (Wolpaw et al., 1983a,c). Reflex change developed over days and weeks, so that a standard 50-day conditioning period (preceded by a 10-day control period) was adopted. The initial results led to the second version of the model, operant conditioning of the monkey triceps surae H-reflex (Wolpaw, 1987), which had two advantages. It eliminated change in muscle spindle sensitivity as the mechanism of the reflex change; and it enabled conditioning in freely moving monkeys (Wolpaw and Herchenroder, 1990). However, the prolonged course of conditioning and the difficulties of primate research constrained mechanistic studies.

**Figure 2:**
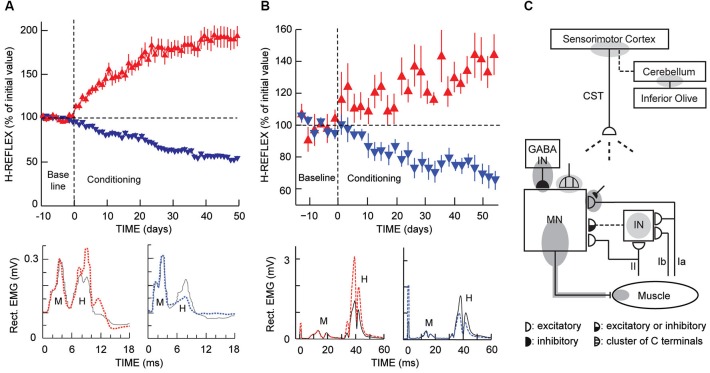
**H-reflex operant conditioning results in rats (A) and humans (B), and the hierarchy of brain and spinal cord plasticity that underlies H-reflex conditioning (C). (A)** As illustrated in Figure 1 (“Rat H-reflex”), in a rat with chronically implanted EMG electrodes and a tibial nerve cuff, the implant wires travel subcutaneously to a head-mounted connector and then through a flexible cable and a commutator to amplifiers and stimulator. The rat moves freely about the cage as soleus muscle activity is monitored 24 h per day. Whenever the absolute (i.e., rectified) value of soleus EMG stays in a specified range for a randomly varying 2.3- to 2.7-s period, a nerve cuff stimulus elicits an M-wave just above threshold and an H-reflex. Top: For the first 10 days (from day –10 to day 0), the rat is exposed to the control mode, in which no reward occurs and the H-reflex is simply measured to determine its initial size. For the next 50 days, it is exposed to the up-conditioning (HRup) or down-conditioning (HRdown) mode, in which a food-pellet reward occurs whenever the H-reflex is above (HRup) or below (HRdown) a criterion value. A rat averages 2000–6000 trials per day, and the criterion is set to provide 500–1000 rewards per day to satisfy the daily requirement (based on body weight). The background EMG and the M-wave stay constant throughout. Successful conditioning (defined as a change of at least 20% in the correct direction) occurs in 75–80% of the rats (the others remain within 20% of their control value). The graphs show average (± SEM) daily H-reflex sizes for 59 successful HRup rats (red upward triangles) and 81 successful HRdown rats (blue downward triangles). In both groups, mode-appropriate change in H-reflex size develops steadily over the 50 days. Bottom: Average absolute post-stimulus EMG for representative days from an HRup rat (left) and an HRdown rat (right) under the control mode (solid) and near the end of HRup or HRdown conditioning (dashed). After conditioning, the H-reflex is larger in the HRup rat and smaller in the HRdown rat, while the background EMG activity and the M-wave have not changed (Updated from Wolpaw, 1997). **(B)** As illustrated in Figure 1 (“Human H-reflex”), EMG activity is monitored in a person with EMG electrodes over the soleus muscle and tibial nerve-stimulating electrodes in the popliteal fossa. The person maintains a natural standing posture facing a screen that displays the current absolute level of soleus EMG in relation to a specified range. Whenever the absolute value of soleus EMG stays in this range for several sec, tibial nerve stimulation elicits an M-wave just above threshold and an H-reflex. Top: For the first six sessions (i.e., baseline sessions, from day −14 to day 0), the person is exposed to the control mode, in which the H-reflex is simply measured to determine its initial size. For the next 24 sessions (i.e., conditioning sessions, days 0–56, three sessions per week), the person is exposed to the HRup or HRdown conditioning mode, in which, after each conditioning trial, the screen provides immediate feedback indicating whether the H-reflex was above (HRup) or below (HRdown) a criterion value. The person completes 225 conditioning trials per session. The background EMG and the M-wave stay constant throughout the sessions. Successful conditioning occurs in about 80% of the people. The graphs show average (± SEM) daily H-reflex sizes for six successful HRup people (red upward triangles) and eight successful HRdown people (blue downward triangles). In both groups, mode-appropriate change in H-reflex size develops steadily over the 24 conditioning sessions. Bottom: Average peri-stimulus EMG from an HRup subject (left) and an HRdown subject (right) for a baseline session (i.e., control mode) (solid) and for the last HRup or HRdown conditioning session (dashed) (A stimulus artifact occurs at 0 ms) (From Thompson et al., 2009). **(C)** A hierarchy of brain and spinal cord plasticity underlies H-reflex conditioning. The shaded ovals indicate the spinal and supraspinal sites of definite or probable plasticity associated with operant conditioning of the H-reflex. “MN” is the motoneuron, “CST” is the main corticospinal tract, “IN” is a spinal interneuron, and “GABA IN” is a GABAergic spinal interneuron. Dashed pathways imply the possibility of intervening spinal interneurons. The monosynaptic and probably oligosynaptic H-reflex pathway from groups Ia, II, and Ib afferents to the motoneuron is shown. Definite (dark gray) or probable (light gray) sites of plasticity include: the motoneuron membrane (i.e., firing threshold and axonal conduction velocity); motor unit properties; GABAergic interneurons; GABAergic terminals and C terminals on the motoneuron; the Ia afferent synaptic connection; terminals conveying oligosynaptic groups I and II inhibition or excitation to the motoneuron; sensorimotor cortex; and cerebellum. As described in the text, the data suggest that the reward contingency acts through the inferior olive to guide and maintain plasticity in the cerebellum that guides and maintains plasticity in sensorimotor cortex that (via the CST) guides and maintains plasticity in the spinal cord that is directly responsible for H-reflex change (Modified from Wolpaw, 2010).

These constraints drove development of the next version, operant conditioning of the soleus H-reflex in the rat (Figure 2A; Chen and Wolpaw, 1995). The rat model reduced the practical difficulties of the work and increased the numbers of animals that could be studied, and thus the questions that could be asked. It remains the primary laboratory tool. More recently, a comparable mouse model has been validated, primarily for its potential application to genomic studies of the conditioning phenomenon (Carp et al., 2006a).

Growing interest in potential clinical applications of reflex conditioning, and demonstration of biceps SSR conditioning in humans (Evatt et al., 1989; Segal and Wolf, 1994; Wolf and Segal, 1996), prompted development of soleus H-reflex conditioning in humans (Figure 2B). While this new version of the model had the three key features of the animal versions (i.e., defined level of pre-stimulus EMG, reward based on reflex size as measured by EMG, and an unchanging reward contingency), it differed in several ways. First, the protocol was not available continuously; conditioning occurred in three one-hour sessions per week, each containing 225 conditioning trials. Thus, over the 8–10 weeks of conditioning, the subjects completed only about 3–5% as many trials as rats did over their 50 days of conditioning. Second, the human H-reflex was elicited and EMG activity was recorded by superficial rather than implanted electrodes. Third, the reward was visual feedback (i.e., a green bar (H-reflex size criterion satisfied) or red bar (H-reflex size criterion not satisfied) on a computer screen) rather than a food pellet. Fourth, a small number of trials at the beginning of each session were control trials, in which the subject was not asked to change the reflex and received no feedback as to reflex size.

The results obtained with these five versions of the model (i.e., monkey biceps SSR, monkey triceps surae H-reflex, rat soleus H-reflex, mouse soleus H-reflex, and human soleus H-reflex) were similar in the proportion of subjects in which conditioning was successful and in the course and final magnitude of reflex change (Wolpaw et al., 1983a; Wolpaw, 1987; Chen and Wolpaw, 1995; Carp et al., 2006b; Thompson et al., 2009). For all versions, conditioning succeeded in 75–80% of the subjects; in the other 20–25%, the reflex remained close to its initial size. Reflex change typically began with a rapid small change in the correct direction, progressed gradually over days and weeks, and appeared to asymptote by 50–60 days (Wolpaw and O’Keefe, 1984; Wolpaw et al., 1994; Chen et al., 2001; Thompson et al., 2009). In rats and monkeys, final reflex size was about 175% of its initial value for up-conditioning and 55% for down-conditioning. In humans, who performed only 3–5% as many trials as the animals, final reflex size averaged 140% for up-conditioning and 69% for down-conditioning. The inter-version and inter-species similarities in course and magnitude of reflex change suggest that mechanistic studies in one version are relevant to understanding the mechanisms in others. Furthermore, the differences in plasticity between successful and unsuccessful animals can provide insight into the substrates of reflex change.

## Mechanistic studies: physiological and anatomical substrates of conditioning

In accord with the original goal, to use reflex operant conditioning to investigate learning and memory, mechanistic studies began in the mid-1980’s and continue at present. They are delineating the spinal cord plasticity associated with conditioning and revealing the role of the brain in producing and maintaining this plasticity.

These studies began with the demonstration that H-reflex conditioning changed the spinal cord: the reflex asymmetry produced by conditioning persisted for at least several days after all supraspinal input was removed (Wolpaw and Lee, 1989). Intracellular studies in primates and rats revealed that successful down-conditioning affected motoneuron firing threshold and reduced axonal conduction velocity (Carp and Wolpaw, 1994; Carp et al., 2001a,b). The positive shift in threshold, plus a small decrease in the primary afferent excitatory postsynaptic potential (EPSP), largely explained the smaller H-reflex (Halter et al., 1995). A change in motoneuron sodium channels was the likely origin of the threshold shift and the reduced conduction velocity (Halter et al., 1995; Wang et al., 2013). In addition, electromicroscopic and immunohistochemical studies found changes in several different synaptic populations on the motoneuron (Carp and Wolpaw, 1994; Halter et al., 1995; Feng-Chen and Wolpaw, 1996; Carp et al., 2001b; Wang et al., 2006; Pillai et al., 2008). Particularly prominent was a marked increase in the number of identifiable GABAergic terminals with successful down-conditioning (Wang et al., 2006); this was accompanied by a corresponding increase in the number of identifiable GABAergic interneurons in the ventral horn (Wang et al., 2009). Furthermore, physiological analyses found change in di- or tri-synaptic pathways contributing to the H-reflex, particularly in up-conditioning (Wolpaw and Chen, 2001), and detected effects on motor unit type (Carp et al., 2001b). Reflex conditioning even affected the contralateral side of the spinal cord (Carp and Wolpaw, 1995).

In summary, H-reflex conditioning produces complex multi-site plasticity in the spinal cord (Figure 2C). While some of these changes appear to underlie the H-reflex change (i.e., *primary plasticity* (Wolpaw and O’Keefe, 1984; Wolpaw, 2010)), others seem to be unrelated (e.g., the change in the contralateral spinal cord (Wolpaw and Lee, 1989; Carp and Wolpaw, 1995; Pillai et al., 2008)). These latter changes are likely to represent *compensatory plasticity* that preserves other behaviors affected by the change in the H-reflex pathway, or *reactive (i.e., downstream) plasticity* due to the changes in ongoing CNS activity produced by primary and compensatory plasticity (Wolpaw and O’Keefe, 1984; Wolpaw, 2010).

Lesion studies of the dependence of H-reflex change on brain-spinal cord connections showed that H-reflex conditioning requires the corticospinal tract and sensorimotor cortex, but does not require other major descending or ascending spinal cord pathways (Chen et al., 2002, 2006a,c; Chen and Wolpaw, 2002). The cerebellum and the inferior olive are essential, at least for down-conditioning (Chen and Wolpaw, 2005; Wolpaw and Chen, 2006; Chen et al., 2012). Since the rubrospinal tract is not needed, cerebellar output to cortex appears to be the cerebellum’s crucial contribution (Chen and Wolpaw, 2005; Wolpaw and Chen, 2006). The effects of specific lesions after down-conditioning has occurred indicates that plasticity in sensorimotor cortex (or closely related areas) is essential for the survival (beyond 5–10 days) of the spinal cord plasticity directly responsible for H-reflex change (Chen et al., 2006a,c), that the cerebellum is essential for the long-term maintenance (beyond 40 days) of this cortical plasticity (Chen and Wolpaw, 2005; Wolpaw and Chen, 2006), and that the cerebellum’s role may depend on cerebellar plasticity that is induced and maintained by climbing fiber input from the inferior olive (Chen et al., 2012). Taken together, these studies showed that H-reflex conditioning depends on a hierarchy of plasticity (i.e., Figure 2C), in which the reward contingency produces plasticity in the brain that guides and maintains the plasticity in the spinal cord that is directly responsible for the H-reflex change (Wolpaw, 2010).

## Therapeutic applications

The work summarized above also explored the impact of H-reflex conditioning on the functioning of the reflex pathway during locomotion and on locomotion itself. In normal rats, the conditioned reflex increases or decreases were evident during locomotion; and the altered H-reflex pathway affected locomotor EMG activity and kinematics. However, conditioning did not disturb important locomotor features, such as right/left symmetry and step-length (Chen et al., 2005). It appeared that compensatory changes in the behavior of other muscles prevented soleus H-reflex conditioning from disturbing normal locomotion (Chen et al., 2011). Nevertheless, this work suggested that H-reflex conditioning might improve locomotion impaired by trauma or disease.

The first exploration of possible therapeutic effects asked whether right soleus H-reflex up-conditioning could improve locomotion in rats in which a lesion of the right lateral column of the spinal cord had weakened right stance and produced an asymmetrical step-cycle (i.e., the rats limped). The step-cycle asymmetry disappeared in up-conditioned rats and did not change in rats in which the H-reflex was simply measured (Chen et al., 2006d). This finding that up-conditioning modified locomotion in spinal cord-injured rats differed from the results in normal rats, in which compensatory plasticity prevented H-reflex conditioning from modifying (i.e., impairing) locomotion (Chen et al., 2011). In rats in which locomotion was already abnormal, H-reflex conditioning aimed at counteracting the deficit improved locomotion.

These results spurred development of the human soleus H-reflex conditioning protocol (Thompson et al., 2009). As noted above, this human protocol had a new feature that was absent in the animal versions. In each conditioning session, it measured 20 control H-reflexes (i.e., elicited without the reward contingency), as well as 225 conditioned H-reflexes. Thus, in addition to demonstrating H-reflex conditioning in humans, it was able to show that this conditioning is the sum of two phenomena: within-session change (i.e., task-dependent adaptation) that appears after 4–6 conditioning sessions and probably reflects immediate change in cortical influence (e.g., on presynaptic inhibition); and gradual across-session change (i.e., long-term change) that begins after 10–12 sessions and probably reflects spinal cord plasticity (Thompson et al., 2009). Task-dependent adaptation affects the H-reflex only during the conditioning protocol. In contrast, long-term change affects the H-reflex continuously and across a wide range of stimulus levels (Thompson et al., 2013a). Thus, it affects other behaviors (e.g., locomotion). The major theoretical and practical significance of this impact on other behaviors is discussed further below.

The development of this human protocol, the evidence that it induces long-term plasticity in the spinal cord pathway, and the evidence of therapeutic efficacy in rats enabled and encouraged an effort to determine whether H-reflex conditioning could improve locomotion in people with chronic incomplete spinal cord injury (SCI; Thompson et al., 2013b). The subjects were people in whom spasticity impaired locomotion; thus down-conditioning of the soleus H-reflex was the logical therapeutic intervention. Over 30 conditioning sessions, the soleus H-reflex decreased in two-thirds of the subjects and remained smaller several months later; in these subjects walking speed increased and right-left symmetry improved, with better EMG modulation across the step-cycle in both legs. In contrast, these improvements did not occur in subjects in whom down-conditioning was not successful or in subjects in whom the H-reflex was simply measured for 30 sessions. Furthermore, beginning about 5 weeks into the conditioning sessions, all of the subjects in whom the H-reflex decreased commented spontaneously that they were walking faster and farther in their daily lives, and several noted less clonus, easier stepping, less arm weight-bearing, and/or other improvements.

These animal and human results indicate that reflex conditioning protocols can improve recovery after chronic incomplete SCI, and possibly in other disorders. Indeed, studies in rats in which the sciatic nerve has been transected and has regenerated suggest that appropriate H-reflex conditioning can improve recovery when the normal Ia afferent connections to motoneurons have been disrupted (Chen et al., 2010).

Operant conditioning protocols designed to change other pathways might also have therapeutic value. Reciprocal inhibition in the spinal cord can be conditioned (Chen et al., 2006b). The motor evoked potential (MEP) in response to transcranial magnetic stimulation can be conditioned; this protocol might be used to improve corticospinal connectivity (Abel et al., 2011; Thompson et al., 2011). The essential attribute of these protocols is that they base reward on the activity in a specific CNS pathway (e.g., the soleus H-reflex pathway, a corticospinal pathway). Thus, they produce “targeted neuroplasticity.” They are both flexible and specific; they can either increase or decrease activity in a specific pathway. Thus, a protocol can be designed to address an individual’s specific deficit. For example, in rats with weak right stance, H-reflex up-conditioning that enhanced the stance-phase soleus burst improved locomotion, while in people with spasticity, down-conditioning that reduced excessive soleus activation through the reflex pathway produced improvement (Chen et al., 2006d; Thompson et al., 2013b). This flexibility and specificity contrast with drug interventions (e.g., botulinum toxin and baclofen), which simply weaken muscles or reflexes and may have undesirable side effects (Dario et al., 2004; Dario and Tomei, 2004; Sheean, 2006; Ward, 2008; Thomas and Simpson, 2012).

Furthermore, the targeted plasticity produced by an appropriate operant conditioning protocol can trigger plasticity elsewhere that has widespread beneficial effects. In people with SCI, the benefits of down-conditioning extended far beyond those attributable to weakening the soleus H-reflex pathway in one leg: locomotor EMG activity improved in proximal and distal muscles of both legs; and people noted improvements in other aspects of motor function (e.g., balance; Thompson et al., 2013b). These widespread benefits are explicable in terms of the “negotiated equilibrium” hypothesis of spinal cord function (Wolpaw, 2010). According to this hypothesis, the brain maintains spinal neurons and synapses in an equilibrium that serves all the behaviors in the current repertoire. When the acquisition of a new behavior (e.g., a larger or smaller H-reflex) changes the spinal cord and disturbs older behaviors, it triggers widespread adaptive changes that arrive at a new equilibrium that serves the expanded repertoire. When an older behavior has been impaired by trauma or disease, the new equilibrium may actually improve it (Chen et al., 2013; Thompson et al., 2013b).

## Conclusion

The importance of basic science as a driver and enabler of clinical progress, and the role of clinical needs in encouraging and justifying basic science, are illustrated by the work reviewed here. When the development of reflex operant conditioning began 35 years ago, the prevailing view of the spinal cord was that it was simply a hard-wired reflex center. The demonstration of reflex operant conditioning helped to overturn this traditional view, and provided a unique model for studying learning and memory in the mammalian CNS. The possibility that the model could have clinical applications did not draw attention for some years. As it has turned out, operant conditioning of spinal cord reflexes is both a valuable laboratory model and a potentially powerful therapeutic method. The experimental accessibility and relative simplicity of the spinal cord, its connection to the brain by well-defined and accessible pathways, its closeness to behavior, and its role (along with the analogous brainstem nuclei) as the final common pathway for all neuromuscular behaviors, make reflex conditioning extremely useful for defining the plasticity underlying a memory, determining how this plasticity affects behavior, and exploring the brain-spinal cord interactions that create and maintain this plasticity. These same attributes underlie its clinical promise. Its ability to target spinal pathways that participate in many important behaviors make reflex operant conditioning a flexible and specific method for addressing functional deficits. Furthermore, its broad beneficial effects are guiding basic studies and generating new thinking about the role of the spinal cord in behavior and about how the many behaviors in an individual’s repertoire are acquired and maintained.

## Conflict of interest statement

The authors declare that the research was conducted in the absence of any commercial or financial relationships that could be construed as a potential conflict of interest.

## References

[B1] Abel B. M. Emore E. Thompson A. K. (2011). Operant up-conditioning of the ankle dorsiflexor motor evoked potential in people with multiple sclerosis. Society for Neuroscience 41st Annual Meeting, Program No. 917.919. Washington, D.C.

[B2] Barbeau H. Rossignol S. (1987). Recovery of locomotion after chronic spinalization in the adult cat. Brain Res. 412, 84–95 10.1016/0006-8993(87)91442-9 3607464

[B3] Barbeau H. (2003). Locomotor training in neurorehabilitation: emerging rehabilitation concepts. Neurorehabil. Neural Repair 17, 3–11 10.1177/0888439002250442 12645440

[B4] Bawa P. Stein R. B. Tatton W. G. (1979). Dynamics of a long-latency reflex pathway in the monkey. Biol. Cybern. 34, 107–110 10.1007/bf00365474 114239

[B5] Carp J. S. Chen X. Y. Sheikh H. Wolpaw J. R. (2001a). Operant conditioning of rat H-reflex affects motoneuron axonal conduction velocity. Exp. Brain Res. 136, 269–273 10.1007/s002210000608 11206290

[B6] Carp J. S. Chen X. Y. Sheikh H. Wolpaw J. R. (2001b). Motor unit properties after operant conditioning of rat H-reflex. Exp. Brain Res. 140, 382–386 10.1007/s002210100830 11681314

[B7] Carp J. S. Tennissen A. M. Chen X. Y. Wolpaw J. R. (2006a). H-reflex operant conditioning in mice. J. Neurophysiol. 96, 1718–1727 10.1152/jn.00470.2006 16837659

[B8] Carp J. S. Tennissen A. M. Chen X. Y. Wolpaw J. R. (2006b). Diurnal H-reflex variation in mice. Exp. Brain Res. 168, 517–528 10.1007/s00221-005-0106-y 16151781

[B9] Carp J. S. Wolpaw J. R. (1994). Motoneuron plasticity underlying operantly conditioned decrease in primate H-reflex. J. Neurophysiol. 72, 431–442 7965025 10.1152/jn.1994.72.1.431

[B10] Carp J. S. Wolpaw J. R. (1995). Motoneuron properties after operantly conditioned increase in primate H-reflex. J. Neurophysiol. 73, 1365–1373 7543942 10.1152/jn.1995.73.4.1365

[B11] Chen X. Chen Y. Chen L. Liu R. Wang Y. Yao L. H. (2012). Inferior olive ablation prevents acquisition and long-term maintenance of soleus H-reflex down-conditioning in rats. Society for Neuroscience 42nd Annual Meeting, Program No. 475.417. New Orleans, LA

[B12] Chen X. Y. Carp J. S. Chen L. Wolpaw J. R. (2002). Corticospinal tract transection prevents operantly conditioned H-reflex increase in rats. Exp. Brain Res. 144, 88–94 10.1007/s00221-002-1026-8 11976762

[B13] Chen X. Y. Carp J. S. Chen L. Wolpaw J. R. (2006a). Sensorimotor cortex ablation prevents H-reflex up-conditioning and causes a paradoxical response to down-conditioning in rats. J. Neurophysiol. 96, 119–127 10.1152/jn.01271.2005 16598062

[B14] Chen X. Y. Chen L. Chen Y. Wolpaw J. R. (2006b). Operant conditioning of reciprocal inhibition in rat soleus muscle. J. Neurophysiol. 96, 2144–2150 10.1152/jn.00253.2006 16807351

[B15] Chen X. Y. Chen L. Wolpaw J. R. (2001). Time course of H-reflex conditioning in the rat. Neurosci. Lett. 302, 85–88 10.1016/s0304-3940(01)01658-5 11290393

[B16] Chen X. Y. Chen Y. Chen L. Tennissen A. M. Wolpaw J. R. (2006c). Corticospinal tract transection permanently abolishes H-reflex down-conditioning in rats. J. Neurotrauma 23, 1705–1712 10.1089/neu.2006.23.1705 17115915

[B17] Chen X. Y. Wolpaw J. R. (1995). Operant conditioning of H-reflex in freely moving rats. J. Neurophysiol. 73, 411–415 7714584 10.1152/jn.1995.73.1.411

[B18] Chen X. Y. Wolpaw J. R. (2002). Probable corticospinal tract control of spinal cord plasticity in the rat. J. Neurophysiol. 87, 645–652 10.1152/jn.00391.2001 11826033

[B19] Chen X. Y. Wolpaw J. R. (2005). Ablation of cerebellar nuclei prevents H-reflex down-conditioning in rats. Learn. Mem. 12, 248–254 10.1101/lm.91305 15930503 PMC1142452

[B20] Chen Y. Chen L. Liu R. Wang Y. Chen X. Y. Wolpaw J. R. (2013).Locomotor impact of beneficial or non-beneficial H-reflex conditioning after spinal cord injury. J. Neurophysiol.. [Epub ahead of print]. 10.1152/jn.00756.2013 24371288 PMC3949309

[B21] Chen Y. Chen L. Wang Y. Wolpaw J. R. Chen X. Y. (2011). Operant conditioning of rat soleus H-reflex oppositely affects another H-reflex and changes locomotor kinematics. J. Neurosci. 31, 11370–11375 10.1523/jneurosci.1526-11.2011 21813696 PMC3156437

[B22] Chen Y. Chen X. Y. Jakeman L. B. Chen L. Stokes B. T. Wolpaw J. R. (2006d). Operant conditioning of H-reflex can correct a locomotor abnormality after spinal cord injury in rats. J. Neurosci. 26, 12537–12543 10.1523/JNEUROSCI.2198-06.2006 17135415 PMC6674902

[B23] Chen Y. Chen X. Y. Jakeman L. B. Schalk G. Stokes B. T. Wolpaw J. R. (2005). The interaction of a new motor skill and an old one: H-reflex conditioning and locomotion in rats. J. Neurosci. 25, 6898–6906 10.1523/jneurosci.1684-05.2005 16033899 PMC6725342

[B24] Chen Y. Wang Y. Chen L. Sun C. English A. W. Wolpaw J. R. (2010). H-reflex up-conditioning encourages recovery of EMG activity and H-reflexes after sciatic nerve transection and repair in rats. J. Neurosci. 30, 16128–16136 10.1523/jneurosci.4578-10.2010 21123559 PMC3005210

[B25] Christensen L. O. Andersen J. B. Sinkjaer T. Nielsen J. (2001). Transcranial magnetic stimulation and stretch reflexes in the tibialis anterior muscle during human walking. J. Physiol. 531, 545–557 10.1111/j.1469-7793.2001.0545i.x 11230526 PMC2278473

[B26] Christensen L. O. Petersen N. Andersen J. B. Sinkjaer T. Nielsen J. B. (2000). Evidence for transcortical reflex pathways in the lower limb of man. Prog. Neurobiol. 62, 251–272 10.1016/s0301-0082(00)00007-1 10840149

[B27] Courtine G. Gerasimenko Y. van den Brand R. Yew A. Musienko P. Zhong H. (2009). Transformation of nonfunctional spinal circuits into functional states after the loss of brain input. Nat. Neurosci. 12, 1333–1342 10.1038/nn.2401 19767747 PMC2828944

[B28] Dario A. Scamoni C. Picano M. Casagrande F. Tomei G. (2004). Pharmacological complications of the chronic baclofen infusion in the severe spinal spasticity. Personal experience and review of the literature. J. Neurosurg. Sci. 48, 177–181 15876986

[B29] Dario A. Tomei G. (2004). A benefit-risk assessment of baclofen in severe spinal spasticity. Drug Saf. 27, 799–818 10.2165/00002018-200427110-00004 15350152

[B30] Di Giorgio A. M. (1929). Persistenza nell’animale spinale, di asimmetrie posturali e motorie di origine cerebellare. Nota I-III. Arch. Fisiol. 27, 519–542

[B31] Di Giorgio A. M. (1942). Azione del cervelletto—neocerebellum—sul tono posturale degli arti e localizzazioni cerebellari nell’animale rombencefalico. Arch. Fisiol. 42, 25–79

[B32] Dietz V. Grillner S. Trepp A. Hubli M. Bolliger M. (2009). Changes in spinal reflex and locomotor activity after a complete spinal cord injury: a common mechanism? Brain 132, 2196–2205 10.1093/brain/awp124 19460795

[B33] Edgerton V. R. Courtine G. Gerasimenko Y. P. Lavrov I. Ichiyama R. M. Fong A. J. (2008). Training locomotor networks. Brain Res. Rev. 57, 241–254 10.1016/j.brainresrev.2007.09.002 18022244 PMC2288528

[B34] Edgerton V. R. Leon R. D. Harkema S. J. Hodgson J. A. London N. Reinkensmeyer D. J. (2001). Retraining the injured spinal cord. J. Physiol. 533, 15–22 10.1111/j.1469-7793.2001.0015b.x 11351008 PMC2278598

[B35] Edgerton V. R. Tillakaratne N. J. Bigbee A. J. de Leon R. D. Roy R. R. (2004). Plasticity of the spinal neural circuitry after injury. Annu. Rev. Neurosci. 27, 145–167 10.1146/annurev.neuro.27.070203.144308 15217329

[B36] Evarts E. V. Tanji J. (1974). Gating of motor cortex reflexes by prior instruction. Brain Res. 71, 479–494 10.1016/0006-8993(74)90992-5 4219749

[B37] Evatt M. L. Wolf S. L. Segal R. L. (1989). Modification of human spinal stretch reflexes: preliminary studies. Neurosci. Lett. 105, 350–355 10.1016/0304-3940(89)90646-0 2594221

[B38] Feng-Chen K. C. Wolpaw J. R. (1996). Operant conditioning of H-reflex changes synaptic terminals on primate motoneurons. Proc. Natl. Acad. Sci. U S A 93, 9206–9211 10.1073/pnas.93.17.9206 8799179 PMC38620

[B39] Frigon A. Rossignol S. (2006). Functional plasticity following spinal cord lesions. Prog. Brain Res. 157, 231–260 10.1016/s0079-6123(06)57016-5 17167915

[B40] Grau J. W. (2013). Learning from the spinal cord: how the study of spinal cord plasticity informs our view of learning. Neurobiol. Learn. Mem. 108C, 155–171 10.1016/j.nlm.2013.08.003 23973905 PMC3946174

[B41] Grey M. J. Ladouceur M. Andersen J. B. Nielsen J. B. Sinkjaer T. (2001). Group II muscle afferents probably contribute to the medium latency soleus stretch reflex during walking in humans. J. Physiol. 534, 925–933 10.1111/j.1469-7793.2001.00925.x 11483721 PMC2278750

[B42] Hall M. (1833). On the reflex function of the medulla oblongata and medulla spinalis. Philos. Trans. R. Soc. Lond. 123, 635–665 10.1098/rstl.1833.0028

[B43] Halter J. A. Carp J. S. Wolpaw J. R. (1995). Operantly conditioned motoneuron plasticity: possible role of sodium channels. J. Neurophysiol. 73, 867–871 7760141 10.1152/jn.1995.73.2.867

[B44] Hammond P. H. (1956). The influence of prior instruction to the subject on an apparently involuntary neuro-muscular response. J. Physiol. 132, 17P–18P 13320395

[B45] Harkema S. J. Hillyer J. Schmidt-Read M. Ardolino E. Sisto S. A. Behrman A. L. (2012). Locomotor training: as a treatment of spinal cord injury and in the progression of neurologic rehabilitation. Arch. Phys. Med. Rehabil. 93, 1588–1597 10.1016/j.apmr.2012.04.032 22920456

[B46] Lee R. G. Murphy J. T. Tatton W. G. (1983). Long-latency myotatic reflexes in man: mechanisms, functional significance, and changes in patients with Parkinson’s disease or hemiplegia. Adv. Neurol. 39, 489–508 6419555

[B47] Lee R. G. Tatton W. G. (1975). Motor responses to sudden limb displacements in primates with specific CNS lesions and in human patients with motor system disorders. Can. J. Neurol. Sci. 2, 285–293 809129 10.1017/s0317167100020382

[B48] Lovely R. G. Gregor R. J. Roy R. R. Edgerton V. R. (1986). Effects of training on the recovery of full-weight-bearing stepping in the adult spinal cat. Exp. Neurol. 92, 421–435 10.1016/0014-4886(86)90094-4 3956672

[B49] Maegele M. Muller S. Wernig A. Edgerton V. R. Harkema S. J. (2002). Recruitment of spinal motor pools during voluntary movements versus stepping after human spinal cord injury. J. Neurotrauma 19, 1217–1229 10.1089/08977150260338010 12427330

[B50] Mendell L. M. (1984). Modifiability of spinal synapses. Physiol. Rev. 64, 260–324 6320234 10.1152/physrev.1984.64.1.260

[B51] Petruska J. C. Ichiyama R. M. Jindrich D. L. Crown E. D. Tansey K. E. Roy R. R. (2007). Changes in motoneuron properties and synaptic inputs related to step training after spinal cord transection in rats. J. Neurosci. 27, 4460–4471 10.1523/jneurosci.2302-06.2007 17442831 PMC6672318

[B52] Pillai S. Wang Y. Wolpaw J. R. Chen X. Y. (2008). Effects of H-reflex up-conditioning on GABAergic terminals on rat soleus motoneurons. Eur. J. Neurosci. 28, 668–674 10.1111/j.1460-9568.2008.06370.x 18657184 PMC2923547

[B53] Rossignol S. Frigon A. Barriere G. Martinez M. Barthelemy D. Bouyer L. (2011). Chapter 16–spinal plasticity in the recovery of locomotion. Prog. Brain Res. 188, 229–241 10.1016/B978-0-444-53825-3.00021-8 21333814

[B54] Segal R. L. Wolf S. L. (1994). Operant conditioning of spinal stretch reflexes in patients with spinal cord injuries. Exp. Neurol. 130, 202–213 10.1006/exnr.1994.1199 7867751

[B55] Sheean G. (2006). Botulinum toxin treatment of adult spasticity: a benefit-risk assessment. Drug Saf. 29, 31–48 10.2165/00002018-200629010-00003 16454533

[B56] Shurrager P. S. Dykman R. A. (1951). Walking spinal carnivores. J. Comp. Physiol. Psychol. 44, 252–262 10.1037/h0059889 14873849

[B57] Thomas A. M. Simpson D. M. (2012). Contralateral weakness following botulinum toxin for poststroke spasticity. Muscle Nerve 46, 443–448 10.1002/mus.23492 22907238

[B58] Thompson A. K. Abel B. M. DeFrancesco E. Lichtman S. W. Pomerantz F. (2011). Operant up-conditioning of the tibialis anterior motor evoked potential in people with incomplete spinal cord injury. International conference of spinal cord medicine and rehabilitation and 37th Annual Meeting of the American Spinal Injury Association, Program No. 71. Washington, D.C.

[B59] Thompson A. K. Chen X. Y. Wolpaw J. R. (2009). Acquisition of a simple motor skill: task-dependent adaptation plus long-term change in the human soleus H-reflex. J. Neurosci. 29, 5784–5792 10.1523/jneurosci.4326-08.2009 19420246 PMC2696311

[B60] Thompson A. K. Chen X. Y. Wolpaw J. R. (2013a). Soleus H-reflex operant conditioning changes the H-reflex recruitment curve. Muscle Nerve 47, 539–544 10.1002/mus.23620 23281107 PMC3608758

[B61] Thompson A. K. Pomerantz F. R. Wolpaw J. R. (2013b). Operant conditioning of a spinal reflex can improve locomotion after spinal cord injury in humans. J. Neurosci. 33, 2365–2375 10.1523/JNEUROSCI.3968-12.2013 23392666 PMC3579496

[B62] Wang Y. Chen Y. Chen L. Wolpaw J. R. Chen X. (2013). Effects of soleus H–reflex conditioning on the motoneuron GABAA receptor, G-protein-activated inwardly-rectifying potassium channel 3.2 and voltage-gated sodium channels. Society for Neuroscience 43rd Annual Meeting, Program No. 645.619. San Diego, CA

[B63] Wang Y. Pillai S. Wolpaw J. R. Chen X. Y. (2006). Motor learning changes GABAergic terminals on spinal motoneurons in normal rats. Eur. J. Neurosci. 23, 141–150 10.1111/j.1460-9568.2005.04547.x 16420424

[B64] Wang Y. Pillai S. Wolpaw J. R. Chen X. Y. (2009). H-reflex down-conditioning greatly increases the number of identifiable GABAergic interneurons in rat ventral horn. Neurosci. Lett. 452, 124–129 10.1016/j.neulet.2009.01.054 19383426 PMC2829943

[B65] Ward A. B. (2008). Spasticity treatment with botulinum toxins. J. Neural Transm. 115, 607–616 10.1007/s00702-007-0833-2 18389166

[B66] Wernig A. Müller S. (1992). Laufband locomotion with body weight support improved walking in persons with severe spinal cord injuries. Paraplegia 30, 229–238 10.1038/sc.1992.61 1625890

[B67] Wolf S. L. Segal R. L. (1996). Reducing human biceps brachii spinal stretch reflex magnitude. J. Neurophysiol. 75, 1637–1646 8727402 10.1152/jn.1996.75.4.1637

[B68] Wolpaw J. R. (1982). Change in short-latency response to limb displacement in primates. Fed. Proc. 41, 2156–2159 7075789

[B69] Wolpaw J. R. (1987). Operant conditioning of primate spinal reflexes: the H-reflex. J. Neurophysiol. 57, 443–459 3559687 10.1152/jn.1987.57.2.443

[B70] Wolpaw J. R. (1997). The complex structure of a simple memory. Trends Neurosci. 20, 588–594 10.1016/s0166-2236(97)01133-8 9416673

[B71] Wolpaw J. R. (2010). What can the spinal cord teach us about learning and memory? Neuroscientist 16, 532–549 10.1177/1073858410368314 20889964

[B72] Wolpaw J. R. Braitman D. J. Seegal R. F. (1983a). Adaptive plasticity in primate spinal stretch reflex: initial development. J. Neurophysiol. 50, 1296–1311 6663327 10.1152/jn.1983.50.6.1296

[B73] Wolpaw J. R. Carp J. S. (1993). Adaptive plasticity in spinal cord. Adv. Neurol. 59, 163–174 8420103

[B74] Wolpaw J. R. Chen X. Y. (2001). Operant conditioning of rat H-reflex: effects on mean latency and duration. Exp. Brain Res. 136, 274–279 10.1007/s002210000609 11206291

[B75] Wolpaw J. R. Chen X. Y. (2006). The cerebellum in maintenance of a motor skill: a hierarchy of brain and spinal cord plasticity underlies H-reflex conditioning. Learn. Mem. 13, 208–215 10.1101/lm.92706 16585796 PMC1409832

[B76] Wolpaw J. R. Herchenroder P. A. (1990). Operant conditioning of H-reflex in freely moving monkeys. J. Neurosci. Methods 31, 145–152 10.1016/0165-0270(90)90159-d 2319815

[B77] Wolpaw J. R. Kieffer V. A. Seegal R. F. Braitman D. J. Sanders M. G. (1983b). Adaptive plasticity in the spinal stretch reflex. Brain Res. 267, 196–200 10.1016/0006-8993(83)91059-4 6860948

[B78] Wolpaw J. R. Lee C. L. (1989). Memory traces in primate spinal cord produced by operant conditioning of H-reflex. J. Neurophysiol. 61, 563–572 2709100 10.1152/jn.1989.61.3.563

[B79] Wolpaw J. R. Maniccia D. M. Elia T. (1994). Operant conditioning of primate H-reflex: phases of development. Neurosci. Lett. 170, 203–207 10.1016/0304-3940(94)90319-0 8058188

[B80] Wolpaw J. R. O’Keefe J. A. (1984). Adaptive plasticity in the primate spinal stretch reflex: evidence for a two-phase process. J. Neurosci. 4, 2718–2724 6502200 10.1523/JNEUROSCI.04-11-02718.1984 PMC6564723

[B81] Wolpaw J. R. Seegal R. F. O’Keefe J. A. (1983c). Adaptive plasticity in primate spinal stretch reflex: behavior of synergist and antagonist muscles. J. Neurophysiol. 50, 1312–1319 6663328 10.1152/jn.1983.50.6.1312

[B82] Wolpaw J. R. Tennissen A. M. (2001). Activity-dependent spinal cord plasticity in health and disease. Annu. Rev. Neurosci. 24, 807–843 10.1146/annurev.neuro.24.1.807 11520919

[B83] Zehr E. P. (2006). Training-induced adaptive plasticity in human somatosensory reflex pathways. J. Appl. Physiol. (1985) 101, 1783–1794 10.1152/japplphysiol.00540.2006 16809627

